# Empathy in Public Safety: Selection Standards, Gender Differences, and Relations with Existing Selection Predictors

**DOI:** 10.3390/jfmk11010032

**Published:** 2026-01-10

**Authors:** Miloš M. Milošević, Nenad Koropanovski, Marko Vuković, Miloš R. Mudrić, Filip Kukić, Irena Ristić, Andreas Stamatis, Milivoj Dopsaj

**Affiliations:** 1 Faculty of Physical Education and Sports Management, Singidunum University, 11000 Belgrade, Serbia; 2 Department of Criminalistics, University of Criminal Investigation and Police Studies, 11000 Belgrade, Serbia; korpan82@gmail.com; 3 Academy for National Security, 11000 Belgrade, Serbia; cipiripi.judo@gmail.com; 4 Faculty of Sport and Physical Education, University in Belgrade, 11000 Belgrade, Serbia; milos.mudric@fsfv.bg.ac.rs (M.R.M.); milivoj.dopsaj@gmail.com (M.D.); 5 Faculty of Physical Education and Sports, University of Banja Luka, 78000 Banja Luka, Bosnia and Herzegovina; filip.kukic@gmail.com; 6 The Faculty of Dramatic Arts, University of Arts in Belgrade, 11000 Belgrade, Serbia; irena.ristic@fdu.bg.ac.rs; 7 Health & Sport Sciences, University of Louisville, Louisville, KY 40292, USA; coach_stam@rocketmail.com; 8 Sports Medicine, University of Louisville, Louisville, KY 40292, USA

**Keywords:** interpersonal reactivity, handgrip strength, body mass index, selection

## Abstract

**Objectives**: This study investigates empathy levels among the public safety personnel and their relationship with current selection indicators (morphological, neuromuscular, and psychological characteristics), highlighting the importance of the topic and its potential for further research. **Methods**: The research was conducted on a sample of 136 police and national security students. The cross-sectional design was applied. Empathy was assessed with the Interpersonal Reactivity Index. Basic morphological characteristics were measured with a portable stadiometer and the InBody 720 device. Neuromuscular characteristics were measured using a handgrip strength test with a sliding device that measures isometric finger flexor force. Psychological characteristics were assessed using the Big Five Plus Two, the Mental Toughness Index, and the Dark Triad Dirty Dozen questionnaires. **Results**: Numerous significant differences between female and male participants, as well as gender-specific correlation patterns, were revealed. Female participants expressed more fantasy (3.23 ± 1.05), empathic concern (3.71 ± 0.75), and personal distress (1.76 ± 0.67) than males (2.84 ± 0.84; 3.37 ± 0.71; 1.5 ± 0.53). Among them, negative correlations of psychological distress with conscientiousness (ρ = −0.66) and mental toughness (ρ = −0.59) stand out. **Conclusions**: This study indicates the possible existence of correlations between empathy, neuromuscular, morphological, and psychological characteristics in public safety personnel of both genders, with gender-specific patterns. Results indicate opportunities for further research aimed at improving the efficiency of the existing selection system.

## 1. Introduction

Empathy can be broadly defined as an individual’s reactions to the perceived experiences of others [1] or as the ability to perceive events from the points of view of others [2]. Empathy refers to both affective and cognitive responses to others and their emotions [3]. The cognitive dimension of empathy can be further divided into perspective-taking (PT), the ability to consider others’ viewpoints, and fantasy (FN), the ability to identify with fictional characters in media or art [1]. On the other hand, the affective dimension of empathy can be further divided into empathic concern (EC), which refers to sympathy for others in need, and personal distress (PD), which refers to self-oriented, negative arousal in response to others’ distress [1]. Although initially formulated by Davis in the 1980s, there is still consensus on the utility of this definition of empathy [1,4,5,6,7]. This definition is largely consistent with the definition of compassion as recognizing suffering, understanding its universality, feeling empathy, tolerating uncomfortable feelings, and the motivation to act to alleviate suffering [8]. Nevertheless, empathy and compassion are closely related yet distinct constructs. Empathy refers to affective and cognitive responsiveness to others’ experiences, while compassion additionally entails motivational and behavioral intentions to alleviate suffering.

Empathy has been widely theorized as one of the most crucial abilities in professions characterized by frequent conflict situations and interpersonal contact [6], such as law enforcement. Empathy is important when interacting with the public under challenging circumstances [9] to understand others’ perceptions of complex situations and, at the same time, to regulate one’s own perceptions [10], both of which are necessary skills for acting professionally. Also, empathy is a necessary ability when working with crime victims [6] to establish and maintain rapport during interviews [5], which is crucial for successful information gathering. The important preventive role of empathy in the criminal sanctions system is also well known and well described [11]. All of these findings indicate that a greater presence of empathy would positively impact the performance of daily duties in public safety.

Furthermore, empathy, as one of the foundations of successful communication, is necessary for successful teamwork [12]. Police students consider empathy the most important ability for gaining tremendous respect and engagement, as well as greater moral value in police work [7]. For similar reasons, Danish police emphasized empathy in the selection and training of officers, which leads to greater community support and trust, more efficient police work, and a more substantial commitment to democratic traditions [13]. This indicates not only that empathy can potentially be important for performing jobs involving stress and contact with people but also that public safety personnel and decision-makers themselves recognize its importance for performing public safety tasks.

In recent years, a growing body of empirical research has examined empathy-focused training interventions in policing, reflecting increased institutional interest in improving police–citizen interactions, procedural justice, and public trust [13,14,15]. Training protocols designed to enhance de-escalation skills among active police officers can elicit emotional engagement relevant to empathic understanding [16], leading to improvements in empathic decision-making patterns [17] and enhanced de-escalation competencies and reduced bias, factors inherently linked to empathic policing practice [18]. These interventions have been associated with improved citizen perceptions, reduced complaints, and more adaptive conflict management, suggesting that empathy is a trainable and operationally relevant competency in law enforcement. However, the existing literature is predominantly intervention- and outcome-oriented, focusing on behavioral changes following training exposure. Much less attention has been paid to baseline empathy levels prior to training, particularly in relation to the selection systems used to recruit police and public safety personnel. Moreover, contemporary empathy-training studies rarely examine how empathy relates to the core selection predictors traditionally emphasized in public safety personnel.

When planning research on empathy in public safety, with the goal of exploring possibilities of integrating empathy into the existing selection and training system, the first step is to choose the assessment instruments. In our case, considering the theoretical definition of empathy [1], it will be the Interpersonal Reactivity Index (IRI), which, in terms of usage [1,3,4,12,19], could be called standard in this field. A recent meta-study showed that, compared to newer instruments, the IRI does not exhibit weaker metric characteristics and has remained one of the most commonly used instruments for assessing empathy over the past 10 years [20].

Moreover, what we currently lack in the field is a detailed description of empathy levels across various public safety personnel as a prerequisite for setting selection standards and cut-off points.

Furthermore, it should be remembered that gender differences in the expression of empathy are well-documented in empirical studies [3,7,21]. Hence, research that takes gender differences into account is necessary to understand the specificities of subpopulations.

It should also be taken into account that the current selection system of public safety workers is based on three dimensions, which are psychological, morphological, and neuromuscular characteristics [13,22,23], whose relations show gender- and occupation-based specific patterns when it comes to comparison of tactical athletes and the general population [24]. Hence, they need to be discussed in more detail in order to understand the context of research in the domain of selection for public safety.

Essential morphological characteristics are important in understanding and predicting occupational performance in public safety [25,26]. They correlate highly with the neuromuscular characteristics of various body parts. Moreover, morphological characteristics are related to personality traits [27,28,29]. That is the reason morphological characteristics form the basis of the selection system for public safety careers.

The handgrip strength (HGS) test has been used as a simple and robust marker of overall body strength [30,31] and health status [32,33,34,35]. It reflects muscular strength and neural activity (i.e., neuromuscular characteristics). A large longitudinal study showed that a 1 kg increase in HGS is associated with a 6% decrease in respiratory disease mortality [35]. Another longitudinal study indicated an inverse dose–response association between incremental levels of handgrip and all-cause cancer mortality in older adults [36]. Furthermore, using a cross-sectional and longitudinal study design, depression was found to be negatively associated with the HGS [37,38]. Moreover, it was reported that HGS is associated with personality traits [39,40,41,42], which are also associated with health outcomes and life expectancy [43,44,45]. Due to the constant exposure to threats in the line of duty, policing ranks among the most high-stress professions [46,47]. Police officers confront a combination of organizational and operational stressors, intensifying the stressful context and impacting their behavior, mental well-being, physical health, and job performance [23,48,49]. Given the robustness of HGS and behavioral determinants, attempts to integrate these principles to improve selection, training, and performance in physically demanding tactical professions have been on the rise [50,51,13,52]. One of the underlying ideas of such an approach is that the relationship between neuromuscular characteristics and behavioral tendencies can serve as indicators of personality, suitable for both job performance in stressful environments and coping with chronic stress. To that end, it is necessary to investigate the possible relationships and effects between indicators and mechanisms of neuromuscular characteristics, namely HGS characteristics and psychological characteristics.

When it comes to psychological characteristics, recent studies suggest that there is more to personality than what can be captured by the Big 5 personality traits and that broadening the domain of personality assessment with mental toughness (MT) and the dark triad (DT) is necessary for valid prediction of stress-related reactions and behaviors [53]. Both MT and DT have a significant role in coping with stress, stress tolerance, and stress reactions [54,55]. Moreover, MT and DT carry valuable information about the perceived stress [53], which is key to understanding and predicting prolonged stress effects and reactions [47]. Given that constant exposure to threats makes policing and national security among the most high-stress professions [46,47], expansion of previous research on the associations between morphological, neuromuscular, and psychological characteristics with MT and DT seems very justified.

The nature and existence of the relationship between morphological, neuromuscular, and psychological characteristics have been documented and explained in previous works across different populations [24,56,57]. Since empathy is a psychological characteristic associated with numerous other psychological characteristics, it is necessary to investigate the relationship between empathy and these characteristics in the public safety personnel. Such research will allow us to explore the existence of a need for integrating empathy into the existing selection and training system. The goals of this study were to explore and describe the level of empathy development among public safety students and the relationship between empathy and psychological, morphological, and neuromuscular characteristics in this specifically selected population with regard to gender-based specificities.

Bearing in mind the results of previous studies, which describe the relationship between psychological, neuromuscular, and morphological factors in various populations [56,57], the relationship between empathy and other mentioned characteristics [1,2,3,4,5,6,7], as well as the way the public safety selection system functions [13,22,23], it was assumed that greater empathy would be associated with a positive selection profile (H1), i.e., with greater development and presence of positive morphological, neuromuscular, and psychological characteristics. It should be emphasized here that PD represents a maladaptive response, so it is expected to correlate in the opposite direction to the other three IRI dimensions. Given the documented gender-specific association of morphological, neuromuscular, and psychological characteristics [24], the second hypothesis (H2) was that there are gender-specific correlations between empathy and psychological, morphological, and neuromuscular characteristics in public safety, i.e., the obtained correlations in female participants will be of a higher degree but in the same direction compared to males.

The results of this study should serve as the first step toward establishing evidence-based selection criteria, as researched by public safety scholars. Furthermore, it paves the way for future research in this domain, offering valuable directions for further exploration.

## 2. Materials and Methods

### 2.1. The Participants

The research was conducted on a sample of first- and second-year students at the University of Criminal Investigation and Police Studies and the Academy for National Security in Belgrade. This sample was selected based on the physical abilities and psychological characteristics needed for the successful completion of training as police and national security officer recruits. The criterion for participation was voluntary registration. The presence of any reported health problems just before and during the study was the exclusion criterion. The sample included 136 participants, of which 54 were females (age = 19.6 ± 0.8 years) and 82 were males (age = 19.9 ± 0.7 years). The gender structure of the sample is similar to that of law enforcement in Serbia [58]. Other descriptive parameters of the subsamples are presented in Table 1. Participants were recruited from the first and second years of study at the University of Criminal Investigation and Police Studies and the Academy for National Security in Belgrade. Participation in the study was strictly voluntary and was not linked in any way to academic evaluation, training assessment, course completion, or future professional status. Recruitment was conducted through general announcements delivered during regular academic activities by staff members not involved in teaching, grading, selection, or evaluation of the participating students. Prior to enrollment, all potential participants received both oral and written information explaining the aims, procedures, potential risks, and benefits of the study. Special attention was given to clarifying the fact that participation or non-participation would have no consequences for students’ academic standing, institutional evaluation, or relationship with instructors or administrators. Students were explicitly informed of their right to refuse to participate or to withdraw from the study at any time, without providing a reason and without any negative consequences. Written informed consent was obtained from all participants before data collection. To minimize potential power dynamics inherent in educational and training institutions, data collection and consent procedures were conducted by researchers who were not directly involved in the participants’ formal education, assessment, or command structure. All collected data were anonymized prior to analysis, and individual results were not accessible to teaching staff, administrators, or selection committees. The study was conducted in accordance with the European Commission’s General Data Protection Regulation and Declaration of Helsinki. The study design was approved by the Ethical Board (number 484-2) of the Faculty of Sport and Physical Education at the University of Belgrade.

### 2.2. Procedures

All testing procedures were conducted within two sessions. During the first session, participants completed the questionnaire, which recorded their socio-demographic status and personality traits and characteristics, with no time limit. The second data collection session was conducted 1 day later and included morphological measurements and the handgrip strength test (HGS). The testing was preceded by a standard warm-up routine, including 5 min of upper-body exercises and 3 min of dynamic exercises that activated the targeted muscle groups, which included the superficial and deep finger flexor muscles (e.g., holding a 10 kg dumbbell, a 5–10 s hang on a pull-up bar, and 3–5 inverted rows). During the last part of the warm-up, each participant performed two HGS trials with a gradual increase in muscle force and two maximally strong and fast trials. The rest between the warm-up trials was 1–2 min, while the rest between the warm-up trials and actual testing procedures was about 5 min. Psychological questionnaires, as well as HGS for dominant and non-dominant hands, were performed in random order.

### 2.3. Morphological Characteristics

Basic body dimensions of body height (BH), body weight (BW), and body mass index (BMI) were used to assess morphological characteristics. BW was measured with a portable stadiometer (Swiss Instruments, Zurich, Switzerland) and the InBody 720 device, according to a standardized procedure [59]. From the obtained values, BMI was calculated.

### 2.4. Neuromuscular Characteristics

HGS was used to assess neuromuscular characteristics. The test was performed using a custom-made sliding device that measures isometric finger flexor force (SMS HG system) and a software system (Isometrics Lite, ver. 3.1.1, Isometrics SMS All4Gym, Belgrade). In the study comparing SMS and Jamar Handgrip dynamometers, inter-rater reliability showed very high agreement (ICC 0.95–0.98) for both instruments, meaning both devices are very reliable for assessing grip strength, though the SMS device showed some lack of concurrent validity (agreement with Jamar) despite high reliability, suggesting they measure similarly but perhaps not identically in absolute values [60]. This is the reason it is widely represented in studies dealing with this topic [49,56,57,60,61] and was therefore selected for this study. The HGS device was attached to the force transducer, which measured the isometric force of the finger flexors. The standard tensiometric probe, with a measurement precision of ±0.01 N, was connected to the force reader. The force-time signal was sampled at 1000 Hz and low-pass filtered (10 Hz) using a fourth-order (zero-phase lag) Butterworth filter [62]. RFD was calculated as the maximal slope of the force-time curve (over the first derivative of the force-time curve) with regard to the force onset [62]. Prior to measurement, the device was calibrated. The onset of the contraction was defined as the point in time when the first derivative of the force-time curve exceeded the baseline by 3% of its maximal value. The device and the procedure have already been used in various research studies with different participant populations, including athletes and tactical athletes [24,60,61,63,64].

Participants performed the HGS test according to previously reported procedures [61,63,64,65]. In short, participants were in a sitting position, holding the measuring device in one hand, with the arm extended alongside the body, while the other arm rested alongside the body. They were not allowed to change position during the test or to lean their hand and the device against the thigh or another solid object.

For the HGS test, participants were instructed to squeeze the device as strongly and as fast as possible. The test was performed twice, with a 2 min rest between trials. Their force output was projected on the screen, and they were verbally encouraged to obtain the best result. From the HGS test, Fmax [N], tFmax [s], RFD [N/s], and tRFD [s] were measured, and they were included in further analyses as sums (S) of values for the dominant and non-dominant hand.

### 2.5. Psychological Characteristics

The Interpersonal Reactivity Index (IRI), Big Five Plus Two (BF + 2), Mental Toughness Index (MTI), and Dark Triad Dirty Dozen (DTDD) questionnaires were used to assess psychological characteristics. To make it easier to compare scores across questionnaires and subscales, scores were calculated as the average of respondents’ answers to all items in a given questionnaire or subscale. This analytical approach was shown to be useful in previous studies [3].

Empathy was assessed using the IRI [1], which consists of 28 items answered on a 5-point Likert scale. The IRI assesses an individual’s empathy through four sub-dimensions: fantasy (Fnt), personal distress (PD), empathic concern (EC), and perspective-taking (PT). The total score and the subscale scores range from 1 (poorly developed) to 5 (significantly developed). The IRI has been widely used in scientific research and clinical practice [4,19] due to its strong psychometric properties [1,3]. One of the many validation studies has shown reliability estimates for IRI with Cronbach’s Alpha (α) ranging from 0.73 to 0.83 [21].

Results of reliability analysis and values of Cronbach’s α for IRI subscales and individual items are presented in Table 2. All the values are at a good and acceptable level. These values did not significantly change when any of the individual items were dropped, with the exception of PD item number 3.

The Big Five personality traits were assessed using the Serbian version of the Big Five Questionnaire. BF + 2 is composed of 70 items, which assess five basic dimensions—neuroticism (Nrc), extraversion (Ext), openness (Opn), conscientiousness (Cns), and aggression (Agr)—as well as two additional dimensions—positive (PV) and negative valence (NV) [66]. Responses to each item are scored on a 5-point Likert scale, ranging from “strongly disagree” (1) to “strongly agree” (5). The total score for each dimension was calculated as the average of the scale scores. The BF + 2 has been widely used in scientific research and clinical practice [67,68] due to its good metric characteristics [66]. In previous studies, reliability estimates for BF + 2 scores ranged from α = 0.81 to α = 0.95 [66].

MT was assessed by the Mental Toughness Index (MTI) [69]. The instrument consists of 8 items, each answered using a 7-point Likert-type scale. The total score ranges from 1 (false 100% of the time) to 7 (true 100% of the time). The construct validity of the MTI has been supported by studies involving participants from various cultures [70,71,72]. In previous studies, reliability estimates for MTI scores were α ≥ 0.86 [70,71,72,73], which is in accordance with the results (Cronbach’s Alpha ≥ 0.81) of the validation study for the assessment of police students [74].

DT traits were assessed using DTDD [75,76], a 12-item measure administered on 7-point Likert-type scales. The DTDD assesses an individual’s overall DT through three socially malevolent traits: Machiavellianism (Mch), psychopathy (Psp), and narcissism (Nrc). The scores range from 1 (poorly present) to 7 (extremely present). In previous studies, reliability estimates for DTDD scores ranged from α = 0.77 to α = 0.84 [75,76], but the validation study for police students [74] showed better results (α ≥ 0.84).

### 2.6. Statistical Analysis

The sample size was determined after applying a power analysis. [77]. For tailed *t*-tests and two-tailed point biserial correlations, with α = 0.05, power (1 − β) = 0.80, and effect size ρ = 0.50, the sample size should be at least 26 participants; for ρ = 0.30, the sample size should be at least 82 participants [77]. For tailed *t*-tests, Wilcoxon, and Mann–Whitney U tests, with a parent distribution of logistic, α = 0.05, power (1 − β) = 0.80, effect size d = 0.55, and an allocation ratio N2/N1 = 1.5, the sample size should be at least 122 participants. Power analyses were performed using G-power 3.1.9.6 (Franz Faul, Universität Kiel, Germany).

All statistical analyses were performed using SPSS 20 (IBM Corp., Armonk, NY, USA). Statistical significance was defined at the 95% probability level (*p* < 0.05), at the 99% probability level (*p* < 0.01), and at the 99.9% probability level (*p* < 0.001).

To assess the level of empathy in the sample, descriptive statistical analyses were initially conducted, including the mean (M), standard deviation (SD), minimum (Min), maximum (Max), and coefficient of variation (CV). The Kolmogorov–Smirnov (KS) test was used to assess the normality of distribution.

In order to reveal gender differences (H1), a non-parametric Mann–Whitney U test was performed. The effect size was calculated according to the following formula:

(1) r = |z|/√N

where r is the effect size, z is the z statistic, and N is the number of observations.

The criterion for evaluation of the effect size was as follows: r < 0.2—small effect, 0.2 < r < 0.8—medium effect, and r > 0.8—significant effect [78].

To examine the relationship between IRI dimensions and psychological, morphological, and neuromuscular characteristics (H2), Spearman’s rank correlation analysis was performed. The effect size of correlation coefficients was defined as follows: weak ρ = 0.20–0.49, moderate ρ = 0.50–0.80, or strong ρ ≥ 0.80 [79].

## 3. Results

Descriptive statistical analysis for the male and female subsamples is presented in Table 1. The Kolmogorov–Smirnov test showed significant deviations from normality for Nrt, NV, and Mch in females and for Nrt, NV, Cns, PD, MT, Mch, DT, and tRFDS in males. According to the CVd, both subsamples showed high variability on the Fnt, PD, Agr, Nrt, NV, Mch, Psc, Nrc, DT, and tFS. Results of bootstrapping of IRI dimensions for male and female subsamples are presented in Table 3.

The non-parametric Mann–Whitney U test revealed significant differences between the female and male subsample with medium effect size in Fnt (U = 1756.50, Z = −2.03, *p* < 0.05, r = 0.27), EC (U = 1692.50, Z = −2.60, *p* < 0.01, r = 0.35), PD (U = 1649.50, Z = −2.53, *p* < 0.05, r = 0.22), Agr (U = 1705.50, Z = −2.26, *p* < 0.05, r = 0.30), MT (U = 1655.50, Z = −2.50, *p* < 0.05, r = 0.33), Psc (U = 1649.50, Z = −2.54 *p* < 0.05, r = 0.33), BMI (U = 1080.00, Z = −5.04, *p* < 0.001, r = −0.71) and tRFDS (U = 1388.00, Z = −3.67, *p* < 0.001, r = 0.51), as well as with large effect size in BH (U = 514.00, Z = −7.57, *p* < 0.001, r = 1.03), BW (U = 504.50, Z = −7.60, *p* < 0.001, r = 1.03), FmaxS (U = 93.50, Z = −9.43, *p* < 0.001, r = 1.30), RFDS (U = 72.00, Z = −9.53, *p* < 0.001, r = 1.29).

Distribution of IRI dimensions within percentile ranks (10, 25, 50, 75, and 90) for the male and female subsamples is presented in Table 4.

Descriptive statistics for the mean PSQ-Op and PSQ-Org, as well as cut-off values calculated from the mean and percentile analysis, are shown in Table 5. Overall, the cut-off range for moderate IRI dimensions is narrower when calculated via percentiles than via mean and standard deviation, making it stricter at the low end but less strict at the high end.

The correlation analysis (Table 6) revealed a weak positive association of PT with Ext, Opn, MT, and tRFDS; and EC with Opn among females, as well as Fnt with Agr, Nrt, and NV; PT with Ext, Opn, Cns, and MT; EC with Ext; and PD with NV, Mch, Nrc, and DT among males. There was also a weak negative association of Fnt with RFDS; PT with Agr and RFDS; EC with Psc, DT, BW, BMI, FmaxS, and RFDS; and PD with Ext, Opn, and PV among females, as well as Fnt with Cns; PT with NV, Psc, DT, and BW; EC with Mch, Psc, and DT; and PD with Opn, PV, Cns, and MT among males. Finally, strong positive correlations were observed in PD with Nrt among both females and males, as well as strong negative correlations in PD with Cns among females and with MT among males.

## 4. Discussion

This study aimed to explore and describe the level of empathy development among students in law enforcement and national security. Additionally, we examined the relationship between empathy and various psychological, morphological, and neuromuscular traits in this specifically selected population.

Our findings, above all, indicate the possibility of a correlation between empathy and morphological, neuromuscular, and psychological characteristics. Most of the obtained correlations, with a few exceptions, are in the expected direction, which generally supports our first hypothesis (H1). When male and female respondents are compared on this issue, it can again be observed that the correlations in females are somewhat more pronounced but in the same direction as in males. These findings generally support our second hypothesis (H2), although the results obtained on this issue are not unanimous.

Before a more in-depth interpretation of the results, it is essential first to examine the characteristics of the sample. According to the descriptive indicators presented in Table 1, Table 3, Table 4 and Table 5 the morphological characteristics and values of all handgrip neuromuscular variables for the sampled participants are above average, even compared to athletes [61,63,80,81,82]. In terms of psychological indicators, the sample is characterized as healthy and resilient to stress compared to the general population [67,68,76]. These findings suggest that the research population has been carefully selected for their superior physical abilities, fitness levels, and psychological profiles [22,23,61]. It is important to consider this fact when discussing the results obtained and drawing conclusions. Also, when drawing conclusions and interpreting the results, it is important to keep in mind that these are first- and second-year students with limited experience in public safety.

Before further discussing the obtained results, it is also necessary to refer to the obtained metric characteristics of the IRI instrument (Table 2). The reliability analysis presented in Table 3 indicates that all subscales exhibit acceptable to good reliability across both the entire sample and the individual subsamples. The only exceptions are EC in female participants and PD in males. Given the characteristics of the sample and the findings from previous validation studies [3], these deviations can be considered expected, minor, and acceptable.

When examining the development of empathy among public safety students, the descriptive indicators (Table 1, Table 3, Table 4 and Table 5) reveal an average presence of EC and PT, which is slightly lower than the average presence of Fnt, and a weak presence of PD. While this distribution of IRI dimensions may seem favorable for future public safety professionals, a comparison with the general population [1,3,21] indicates that our sample does not stand out as one might expect from such a selectively chosen group.

This conclusion is further supported by comparisons with Swedish police students’ findings [6]. A detailed analysis of the descriptive indicators in the distribution of IRI scores (Table 3, Table 4 and Table 5) leads to a similar conclusion. While a significant portion of our sample demonstrates exceptional empathy, a considerable number have average or below-average IRI scores, particularly among males.

Descriptive analyses (Table 1, Table 3, Table 4 and Table 5) and analyses of variance revealed expected gender differences [1,3,21]. Specifically, female participants demonstrated higher levels of development and presence across all IRI subscales. The observed differences in empathy and other measured characteristics between female and male participants are consistent with previous studies’ results [1,3,21,61,63,80,81,82]. Females tend to be smaller and physically weaker than males, exhibit higher levels of empathy, and show fewer tendencies toward antisocial behavior. In contrast, males are generally more physically robust and demonstrate greater MT development.

One unexpected finding is the greater level of aggressiveness observed in females, which diverges from previous research. This observation may be attributed to the characteristics of the specific sample and the recruitment, but further research is necessary to draw definitive conclusions.

Considering all of the above, it seems reasonable to establish separate selection standards for female and male students in public safety. Our findings suggest that establishing cut-off points using percentiles will provide a better distribution of scores in the current population, while if more stringent selection criteria are required, establishing cut-off points using mean and standard deviation would be preferable. However, this is only an initial study; the validity of both approaches needs to be further tested in future studies that will more clearly demonstrate the practical value of the guidelines.

Before proceeding to the discussion of the results of the correlation analysis, it should be noted that the sample was designed based on a power analysis that takes into account the medium effect and that correlations that are less than 0.5 do not meet the given criterion.

Consistent with expectations [3,21], correlation analyses (Table 6) identified positive relationships between empathy components, such as EC and PT, and prosocial and positive behavioral tendencies. Conversely, negative correlations were observed with the dimensions of Agr, Nrt, and DT. Also, as expected, the PD dimension showed relationships in the opposite direction. Furthermore, the Fnt dimension, particularly among males, showed positive correlations with Agr, Nrt, and NV and a negative correlation with Cns. These findings align with previous validation studies [3] and highlight the need for more in-depth research into this dimension.

However, when examining the relationship between empathy and morphological and neuromuscular characteristics, previous research [27,28,29,39,40,41,83,84,85] has established links between BMI and hand grip strength with various behavioral indicators. A similar relationship was anticipated for empathy. The expected results were found for females, while for males, the only significant finding was a negative correlation between BW and PT. Overall indicators of physical strength, ability, and fitness were negatively associated with empathy in females, with RFD being particularly noteworthy. Because our study was designed for medium-effect correlations, we must accept our first hypothesis. However, these data suggest the possibility that empathy and physical strength do not go hand in hand, especially among females. If we add to this finding the previously observed greater aggressiveness of female participants, the possibility for future research emerges, because it could provide us with more information about how to select effective future public safety workers.

Although this study provided valuable insights, it has several limitations. The intentional selection of a specific group of police and national security students restricts the generalizability of the findings. Namely, the sample consists of first- and second-year students from specific Serbian institutions, which may not generalize to broader public safety personnel or experienced officers. As a result, the conclusions may not be applicable to other public safety cohorts or to the broader population, given the sample’s focused nature. This specificity limits our ability to make broad conclusions about attitudes and behaviors in a larger context, particularly when influenced by socio-demographic factors. Additionally, since this study is correlational, it is important to clarify that no causal relationships can be established between the variables examined, as correlation does not imply causation. Also, in psychological assessment, self-assessment scales were used that do not necessarily represent objective measures of psychological characteristics, and there is also the possibility of expecting certain types of behavior from members of public security, leading them to give socially desirable responses.

The results obtained can have implications for further research on empathy in public safety. They provide a basis for asking questions that are necessary for improving candidate selection processes, especially regarding psychological assessments. While definitive answers would require longitudinal studies, our findings suggest that it may be beneficial to investigate candidates’ empathy levels, job performance, and responses to stress. This exploration could have potential in enhancing the quality of public safety workers’ selection and promoting the well-being of those in these roles.

## Figures and Tables

**Table 1:** Descriptive statistical analysis for the male and female subsamples.

	Female (n = 54)						Male (n = 82)					
	Min	Max	M	SD	CV	KS Z	Min	Max	M	SD	CV	KS Z
Fnt *	1	5	3.23	1.05	0.33	0.62	1	5	2.84	0.84	0.30	1.04
PT	2	5	3.89	0.66	0.17	0.62	2	5	3.8	0.66	0.17	0.83
EC **	1.57	5	3.71	0.75	0.20	0.77	1.57	5	3.37	0.71	0.21	0.57
PD *	1	4.57	1.76	0.67	0.38	0.97	1	3.57	1.50	0.53	0.35	1.75 **
Agr *	1	4.1	2.28	0.77	0.34	0.79	1	4.3	1.99	0.79	0.40	1.16
Ext	2.5	5	4.13	0.6	0.15	1.08	1.4	5	4.17	0.69	0.17	1.21
Nrt	1	4.3	1.59	0.74	0.47	1.56 *	1	3.4	1.51	0.68	0.45	2.13 ***
NV	1	2.3	1.32	0.36	0.27	1.54 *	1	3.7	1.48	0.62	0.42	1.97 **
Opn	3	4.9	4.1	0.53	0.13	0.79	2.6	5	4.15	0.55	0.13	1.14
PV	1.8	5	3.69	0.74	0.20	0.77	1.6	5	3.64	0.74	0.20	0.81
Cns	2.4	5	4.39	0.61	0.14	1.15	2.4	5	4.35	0.73	0.17	1.78 **
MT *	3.75	7	6.21	0.6	0.10	0.69	5.38	7.13	6.46	0.49	0.08	1.68 **
Mch	1	4.75	1.75	0.99	0.57	1.64 *	1	6.17	2.18	1.31	0.60	1.67 **
Psc *	1	4.5	1.86	0.96	0.52	1.37	1	7	2.4	1.32	0.55	1.29
Nrc	1	6	3.07	1.41	0.46	0.85	1	6.25	3.18	1.55	0.49	0.87
DT	1	4.33	2.21	0.85	0.38	1.00	1	6.17	2.55	1.17	0.46	1.38 *
BH ***	1.62	1.85	1.71	0.06	0.04	0.68	1.65	1.94	1.81	0.06	0.03	0.77
BW ***	51	104	65.05	8.68	0.13	0.97	54.6	136.7	78.91	10.46	0.13	0.88
BMI ***	18.96	36.85	22.3	2.89	0.13	1.22	18.89	37.08	24	2.41	0.10	1.13
FmaxS ***	456	791	605.31	81.9	0.14	0.92	602	1427	993.83	180.65	0.18	0.49
RFDS ***	2390	5674	3811	653.26	0.17	0.32	3870	9507	6519.94	1159.18	0.18	0.57
tFS	0.75	4.75	1.88	0.67	0.36	0.68	0.62	5.66	1.98	0.9	0.45	1.08
tRFDS ***	0.19	0.43	0.25	0.04	0.16	1.28	0.2	0.46	0.24	0.04	0.17	2.16 ***

Note: Min—minimum, Max—maximum, M—mean, SD—standard deviation, CV—coefficient of variation, KS Z—Kolmogorov–Smirnov Z score, Fnt—fantasy, PT—perspective tacking, EC—empathetic concern, PD—personal distress, Agr—aggression, Ext—extraversion, Nrt—neuroticism, NV—negative valence, Opn—openness, PV—positive valence, Cns—conscientiousness, MT—mental toughness, Mch—Machiavellianism. Psc—psychopathy. Nrc—narcissism, DT—dark triad, BH—body height [cm], BW—body weight [kg], BMI—body mass index [kg/m^2^], Fmax—the maximal force [N], RFD—maximal rate of force development [N/s], tF—time required to reach the maximal force [s], tRFD—the time required to reach the maximal rate of force development, S—sum of dominant and non-dominant hand, * *p* < 0.05, ** *p* < 0.01, *** *p* < 0.001.

**Table 2:** Reliability analysis for IRI subscales and individual items.

	All (N = 613)				Female (n = 54)				Male (n = 82)			
	Fnt	PT	EC	PD	Fnt	PT	EC	PD	Fnt	PT	EC	PD
Cronbach’s α	0.82	0.72	0.70	0.79	0.87	0.75	0.73	0.65	0.75	0.70	0.65	0.84
Items	Cronbach’s α if Item Deleted											
1	0.81	0.70	0.62	0.75	0.86	0.72	0.61	0.57	0.73	0.69	0.60	0.81
2	0.77	0.67	0.69	0.77	0.35	0.70	0.70	0.63	0.71	0.65	0.66	0.82
3	0.83	0.69	0.71	0.83	0.87	0.75	0.77	0.72	0.78	0.64	0.71	0.87
4	0.81	0.74	0.62	0.75	0.87	0.77	0.65	0.55	0.74	0.73	0.63	0.81
5	0.77	0.68	0.70	0.75	0.85	0.72	0.72	0.58	0.67	0.65	0.57	0.80
6	0.78	0.67	0.66	0.76	0.83	0.66	0.73	0.63	0.70	0.68	0.68	0.80
7	0.78	0.66	0.65	0.77	0.86	0.71	0.68	0.57	0.68	0.62	0.59	0.83

Note: Fnt—fantasy, PT—perspective tacking, EC—empathetic concern, PD—personal distress.

**Table 3:** Bootstrapping of interpersonal reactivity dimensions for male and female subsamples.

		Female (n = 54)					Male (n = 82)				
		Statistic	Bias	SE	95% Confidence Interval		Statistic	Bias	SE	95% Confidence Interval	
					Lower	Upper				Lower	Upper
Fnt	M	3.23	−0.01	0.14	2.95	3.50	2.84	0.00	0.09	2.67	3.03
	SD	1.05	−0.01	0.08	0.88	1.19	0.84	−0.01	0.06	0.72	0.96
PT	M	3.89	0.00	0.09	3.73	4.07	3.80	0.00	0.07	3.65	3.94
	SD	0.66	−0.01	0.06	0.52	0.78	0.66	−0.01	0.05	0.56	0.75
EC	M	3.71	0.00	0.10	3.51	3.90	3.37	0.00	0.08	3.21	3.53
	SD	0.75	−0.01	0.06	0.63	0.87	0.71	−0.01	0.05	0.60	0.81
PD	M	1.76	0.00	0.09	1.59	1.95	1.50	0.00	0.06	1.40	1.62
	SD	0.69	−0.02	0.11	0.47	0.88	0.53	−0.01	0.06	0.42	0.65

Note: M—mean, SD—standard deviation, SE—standard error, Fnt—fantasy, PT—perspective tacking, EC—empathetic concern, PD—personal distress.

**Table 4:** Percentiles for interpersonal reactivity dimensions for male and female subsamples.

	Percentiles	Fnt	PT	EC	PD
Female (n = 54)	10	2.00	3.21	2.71	1.00
	25	2.39	3.43	3.11	1.25
	50	3.14	3.86	3.86	1.71
	75	4.14	4.43	4.32	2.14
	90	4.71	4.79	4.57	2.64
Male (n = 82)	10	1.57	3.00	2.43	1.00
	25	2.11	3.43	2.96	1.11
	50	3.00	3.71	3.43	1.43
	75	3.43	4.29	3.86	1.71
	90	3.71	4.81	4.29	2.39

Note: Fnt—fantasy, PT—perspective tacking, EC—empathetic concern, PD—personal distress.

**Table 5:** Descriptive statistics and cut-off values for the interpersonal reactivity dimensions for male and female subsamples.

		Female (n = 54)		Male (n = 82)	
	Statistics	Value	Cut-Off	Value	Cut-Off
Fnt	M ± SD	3.23 ± 1.05	Low < 2.2	2.84 ± 0.84	Low = < 2.0
			Moderate = 2.2–4.3		Moderate = 2.0–3.7
			High > 4.3		High > 3.7
	Percentiles	33rd = 2.7	Low < 2.7	33rd = 2.6	Low < 2.6
		67th = 3.7	Moderate = 2.7–3.7	67th = 3.3	Moderate = 2.6–3.3
			High > 3.7		High > 3.3
PT	M ± SD	3.89 ± 0.66	Low < 3.2	3.8 ± 0.66	Low = < 3.1
			Moderate = 3.2–4.6		Moderate = 3.1–4.5
			High > 4.6		High > 4.5
	Percentiles	33rd = 3.6	Low < 3.6	33rd = 3.6	Low < 3.6
		67th = 4.1	Moderate = 3.6–4.1	67th = 4.1	Moderate = 3.6–4.1
			High > 4.1		High > 4.1
EC	M ± SD.	3.71 ± 0.75	Low < 3.0	3.37 ± 0.71	Low = < 2.7
			Moderate = 3.0–4.5		Moderate = 2.7–4.1
			High > 4.5		High > 4.1
	Percentiles	33rd = 3.3	Low < 3.3	33rd = 3.1	Low < 3.1
		67th = 4.1	Moderate = 3.3–4.1	67th = 3.6	Moderate = 3.1–3.6
			High > 4.1		High > 3.6
PD	M ± SD	1.76 ± 0.67	Low < 1.1	1.5 ± 0.53	Low = < 1.0
			Moderate = 1.1–2.4		Moderate = 1.0–2.0
			High > 2.4		High > 2.0
	Percentiles	33rd = 1.4	Low < 1.4	33rd = 1.1	Low < 1.1
		67th = 1.9	Moderate = 1.4–1.9	67th = 1.6	Moderate = 1.1–1.6
			High > 1.9		High > 1.6

Note: M—mean, SD—standard deviation, Fnt—fantasy, PT—perspective tacking, EC—empathetic concern, PD—personal distress.

**Table 6:** Correlation analysis of IRI subscales with psychological, morphological, and neuromuscular characteristics for male and female subsamples.

	Female (n = 54)				Male (n = 82)			
	Fnt	PT	EC	PD	Fnt	PT	EC	PD
Agr	0.03	−0.37 **	−0.25	0.04	0.33 **	−0.21	−0.14	0.12
Ext	0.17	0.31 *	0.26	−0.33 *	−0.04	0.29 **	0.29 **	−0.12
Nrt	0.25	−0.26	0.06	0.62 **	0.38 **	−0.10	0.08	0.50 **
NV	0.12	−0.17	−0.23	0.26	0.30 **	−0.22 *	−0.18	0.33 **
Opn	0.41 **	0.40 **	0.34 *	−0.37 **	0.19	0.31 **	0.11	−0.32 **
PV	0.16	0.19	−0.03	−0.43 **	0.21	0.08	0.01	−0.23 *
Cns	−0.22	0.29 *	0.01	−0.66 **	−0.24 *	0.24 *	0.11	−0.46 **
MT	−0.06	0.42 **	0.02	−0.59 **	0.01	0.25 *	0.02	−0.55 **
Mch	−0.02	−0.11	−0.25	0.20	0.11	−0.17	−0.32 **	0.25 *
Psc	−0.07	−0.20	−0.36 **	−0.06	−0.01	−0.37 **	−0.32 **	0.13
Nrc	−0.14	−0.08	−0.23	−0.07	0.19	−0.14	−0.10	0.29 **
DT	−0.12	−0.13	−0.37 *	−0.03	0.16	−0.24 *	−0.26 *	0.26 *
BH	−0.05	−0.04	−0.15	−0.08	−0.01	−0.11	0.00	−0.17
BW	−0.20	−0.08	−0.36 **	−0.06	−0.11	−0.25 *	−0.11	−0.12
BMI	−0.24	−0.06	−0.35 *	−0.03	−0.18	−0.19	−0.13	−0.07
FmaxS	−0.25	−0.13	−0.34 *	−0.09	0.05	−0.10	−0.14	−0.05
RFDS	−0.28 *	−0.31 *	−0.45 **	−0.03	0.09	−0.13	−0.11	−0.05
tFS	−0.08	0.08	0.04	−0.08	0.02	0.07	0.03	−0.22
tRFDS	0.20	0.34 *	0.18	−0.09	−0.02	0.10	−0.08	−0.03

Note: Fnt—fantasy, PT—perspective tacking, EC—empathetic concern, PD—personal distress, Agr—aggression, Ext—extraversion, Nrt—neuroticism, NV—negative valence, Opn—openness, PV—positive valence, Cns—conscientiousness, MT—mental toughness, Mch—Machiavellianism. Psc—psychopathy. Nrc—narcissism, DT—dark triad, BH—body height [cm], BW—body weight [kg], BMI—body mass index [kg/m^2^], Fmax—the maximal force [N], RFD—maximal rate of force development [N/s], tF—time required to reach the maximal force [s], tRFD—the time required to reach the maximal rate of force development, S—sum of dominant and non-dominant hand, * *p* < 0.05, ** *p* < 0.01.

## Data Availability

The data presented in this study are not publicly available due to ethical, institutional, and national security considerations related to the study population. De-identified and aggregated datasets supporting the findings of this study may be made available from the corresponding author upon reasonable request for academic and research purposes, subject to approval by the relevant institutional ethics committee and compliance with applicable data protection regulations.
